# Defective response inhibition and collicular noradrenaline enrichment in mice with duplicated retinotopic map in the superior colliculus

**DOI:** 10.1007/s00429-014-0745-5

**Published:** 2014-03-20

**Authors:** Chantal Mathis, Elise Savier, Jean-Bastien Bott, Daniel Clesse, Nicholas Bevins, Dominique Sage-Ciocca, Karin Geiger, Anaïs Gillet, Alexis Laux-Biehlmann, Yannick Goumon, Adrien Lacaud, Vincent Lelièvre, Christian Kelche, Jean-Christophe Cassel, Frank W. Pfrieger, Michael Reber

**Affiliations:** 1 Laboratory of Adaptative and Cognitive Neurosciences, CNRS, University of Strasbourg UMR 7364, 67000 Strasbourg, France; 2 Molecular Neurobiology Laboratory, The Salk Institute, La Jolla, San Diego, CA 92037 USA; 3 Department of Neurosciences, University of California, La Jolla, San Diego, CA 92039 USA; 4 Institute of Cellular and Integrative Neurosciences, CNRS UPR 3212, University of Strasbourg, 5, rue blaise Pascal, 67084 Strasbourg, France; 5 Chronobiotron, UMS 3415, CNRS, 67084 Strasbourg, France

**Keywords:** Retinotopy, Visual system, EphA signaling, Superior colliculus, Noradrenaline, Response inhibition, Attention-deficit disorders

## Abstract

The superior colliculus is a hub for multisensory integration necessary for visuo-spatial orientation, control of gaze movements and attention. The multiple functions of the superior colliculus have prompted hypotheses about its involvement in neuropsychiatric conditions, but to date, this topic has not been addressed experimentally. We describe experiments on genetically modified mice, the Isl2-EphA3 knock-in line, that show a well-characterized duplication of the retino-collicular and cortico-collicular axonal projections leading to hyperstimulation of the superior colliculus. To explore the functional impact of collicular hyperstimulation, we compared the performance of homozygous knock-in, heterozygous knock-in and wild-type mice in several behavioral tasks requiring collicular activity. The light/dark box test and Go/No-Go conditioning task revealed that homozygous mutant mice exhibit defective response inhibition, a form of impulsivity. This defect was specific to attention as other tests showed no differences in visually driven behavior, motivation, visuo-spatial learning and sensorimotor abilities among the different groups of mice. Monoamine quantification and gene expression profiling demonstrated a specific enrichment of noradrenaline only in the superficial layers of the superior colliculus of Isl2-EphA3 knock-in mice, where the retinotopy is duplicated, whereas transcript levels of receptors, transporters and metabolic enzymes of the monoaminergic pathway were not affected. We demonstrate that the defect in response inhibition is a consequence of noradrenaline imbalance in the superficial layers of the superior colliculus caused by retinotopic map duplication. Our results suggest that structural abnormalities in the superior colliculus can cause defective response inhibition, a key feature of attention-deficit disorders.

**Electronic supplementary material:**

The online version of this article (doi:10.1007/s00429-014-0745-5) contains supplementary material, which is available to authorized users.

## Introduction

The superior colliculus (SC) is a midbrain structure that integrates sensory inputs from multiple modalities (Wallace et al. 1993; Holmes and Spence 2005; May 2006) and plays a central role in visuo-spatial orientation, attention and sensorimotor processing (Stein 1984; May 2006; Gandhi and Katnani 2011). Defects in SC function have been associated with a number of neuropathological and neuropsychiatric disorders including epilepsy (Ross and Coleman 2000), schizophrenia (Fuentes 2001) and autism spectrum disorder (ASD) (Kleinhans et al. 2011). Recently, collicular hyperstimulation has been proposed to underlie attention-deficit/hyperactivity disorder (ADHD) symptoms, especially the impulsivity and distractibility associated with the disorder (Overton 2008; Miller 2009; Dommett et al. 2009). However, direct experimental evidence for such a link remains elusive.

The SC presents a particular feature, namely the topographic organization of its sensory inputs (Sperry 1963; Lemke and Reber 2005; May 2006). Axons of retinal ganglion cells (RGCs) project to the superficial layers of the SC along spatial axes reflecting their position along corresponding axes in the retina (the retino-collicular map). Layer V neurons of the V1 cortex also project in a topographic manner to the superficial layers of the SC, the cortico-collicular map, which is in register with the retino-collicular map (May 2006; Triplett et al. 2012). This creates a topographic representation of the visual field in the superficial layers of the SC, also called retinotopy. Auditory and somatosensory afferents projecting to deep layers of the SC are also aligned with the visual maps (Meredith and Stein 1985; King et al. 1998; May 2006) enhancing perception of salient stimuli and influencing decision and overt behavior (Stein et al. 2009).

**Fig. 1:**
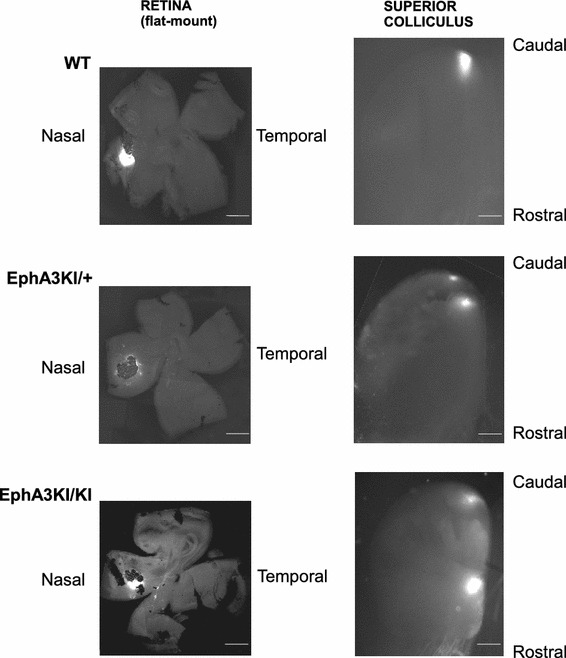
Topographic retino-collicular projections in WT and Isl2-EphA3KI animals. Micrographs illustrate nasal 1,1′-dioctadecyl-3,3,3′3′-tetramethylindocarbocyanine perchlorate (Dil) injections in P8 retinas and the corresponding termination zone(s) in the SC. *Top* an injection in nasal WT retina leads to a single caudal termination zone in the SC. *Middle* an injection in a nasal EphA3KI/+ retina leads to two caudal termination zones in the SC. *Bottom* an injection in a nasal EphA3KI/KI retina leads to two distant termination zones in the SC. *Scale bars* 1 mm

We took advantage of a specific disruption of the retinotopy in the superficial layers of the SC that has been observed in the Isl2-EphA3 knock-in mice (Fig. 1; Brown et al. 2000). In this mouse model, the EphA3 tyrosine kinase receptor, which acts as a guidance molecule during map formation, is over-expressed by a subset of RGCs. This leads to a well-characterized duplication of the retino-collicular and cortico-collicular maps along the anterior–posterior axis of the SC. Over-expression of the EphA3 receptor neither affects retinal organization and integrity, nor the topography of collicular somatosensory inputs (Brown et al. 2000; Reber et al. 2004; Triplett et al. 2009; Bevins et al. 2011; Triplett et al. 2012). The duplicated visual maps are functional as single visual stimuli trigger the activation of two distinct areas in the SC (Triplett et al. 2009). Unlike other mouse models that target Eph/ephrin signaling (Dottori et al. 1998; Feldheim et al. 2000; Feldheim 2004), the genetic modification in the Isl2-EphA3 knock-in mice affects only a subset of RGCs and does not affect other structures in the brain (Brown et al. 2000; Reber et al. 2004, Thaler et al. 2004).

To determine if hyperstimulation of the SC, due to duplication of the retinotopic projections, influences collicular-related behavior, wild-type (WT), heterozygous (EphA3^KI/+^) and homozygous (EphA3^KI/KI^) Isl2-EphA3KI mice were subjected to a series of well-established behavioral tests. As a first approach, we tested general visual ability (cliff test, optokinetic reflex, Morris water maze with visible platform) as the effects of disrupted EphAs gradients in the RGCs and duplicated retinotopy in the SC on visual perception have never been described before. We then focused on general sensorimotor (locomotor activity, circadian rhythmicity, light/dark box test) and integrative features (beam walking test) and on collicular-related behavior, especially visuo-spatial orientation and memory (Morris water maze with hidden platform) and response inhibition (Go/No-Go task). Our results show that EphA3^KI/KI^ mutant mice exhibit defective response inhibition when compared to WT or EphA3^KI/+^ littermates. Visual acuity, sensorimotor activity, visuo-spatial learning, motivation and memory were similar in the different genotypes. Molecular characterization demonstrated elevated noradrenaline levels in the superficial layers of the SC in EphA3^KI/KI^ animals where the retinotopy is duplicated. Expression levels of receptors, transporters and enzymes of the monoaminergic signaling pathway were similar to WT littermates. Interestingly, these changes resemble specific symptoms of the adult and predominantly inattentive-type of ADHD patients (Diamond 2005; Biederman and Faraone 2005).

## Materials and methods

### Animals

Mice were bred and housed in our mouse facility (Chronobiotron, UMS 3415, CNRS, Strasbourg) and tested during the light phase (ZT2–ZT10) of their light/dark cycle except for indicated experiments. All procedures were in accordance with national (council directive 87/848, October 1987) and European community (2010/63/EU) guidelines. Official agreement numbers for animal experimentation were 67-292 for CM, 67-215 for J-CC and 67-358 for KG, AG was under their responsibility. Mice were genotyped by PCR of genomic DNA from tail biopsies as described previously (Reber et al. 2004). Four- to seven-month-old male littermates of each genotype (EphA3^KI/KI^, EphA3^KI/+^ and WT) on a mixed genetic background (C57/Bl6 × 129Sv/J) were subjected to behavioral tests and molecular analyses. Standard laboratory rodent food and water were available ad libitum throughout all experiments, except for the Go/No-Go task, for which all mice were kept at 85 % of their free-feeding weight.

### Behavioral tests

Three distinct cohorts of 4- to 7-month-old WT, EphA3^KI/+^ and EphA3^KI/KI^ males littermates were characterized using fixed sequences of test ranging as much as possible from the least to the most invasive test. Inter-test intervals (ITI) varied along the sequences to limit order effect. The first cohort of 4- to 7-month-old males littermates (*n* = 6–9 per group) was first tested in the light/dark box test (Boeuf et al. 2009) (ITI 5 days) and then only in the Go/No-Go task (Meziane et al. 1993). The second cohort of 4- to 7-month-old males littermates (*n* = 7 per group) was dedicated to sensorimotor evaluations. They were first tested for circadian wheel running activity (Mendoza et al. 2008) and general locomotor activity (Yassine et al. 2013) (ITI 15 days) followed by the Morris water maze paradigm (Moreau et al. 2008) (ITI 15 days), the beam walking test (Moreau et al. 2008) (ITI 3 days) and the visual cliff test (Gibson and Walk 1960) (ITI 21 days). The optokinetic reflex (Douglas et al. 2005) was studied on a third cohort of 4-month-old (*n* = 7–10) male littermates. Detailed descriptions can be found in Online resource 1.

### Molecular analysis

Transcript levels were analyzed by semi-quantitative PCR and monoamine levels were measured by high-pressure liquid chromatography as described in the Online resource 1.

### Statistical analysis

Unless otherwise indicated, data were analyzed by analysis of variance with repeated measure factors to study interactions between genotype and side, trial, day, 15-min block, quadrant, runway (rANOVA). All statistical outcomes were confirmed by a Kruskal–Wallis test applied on the light–dark single factors or within each repeated measure, as group sizes in behavioral studies were relatively small. When required, post hoc analyses were performed with the Newman–Keuls (NK) multi-comparison test (Statistica 8.0; Statsoft, Inc., Tulsa, OK). The time spent in the goal quadrant of the water maze was compared to the 15-s chance value by means of a *t* test. The 15-s chance value corresponds to the time spent for random search in four quadrants during the 60 s probe test. All behavioral data are expressed as mean ± standard error of the mean (SEM). HPLC and qPCR data were analyzed using the non-parametric Kruskal–Wallis (KW) test. All expression data are represented using boxplots (min, q1, median, q3, max).

## Results

The functional contribution of the SC in specific behavior has been investigated in a variety of experiments, including electrophysiological recording, inactivation and lesion approaches (Binns 1999; Huberman and Niell 2011) but little has been done at a more integrated level in animal models with congenital defects.

### Visual acuity

**Fig. 2:**
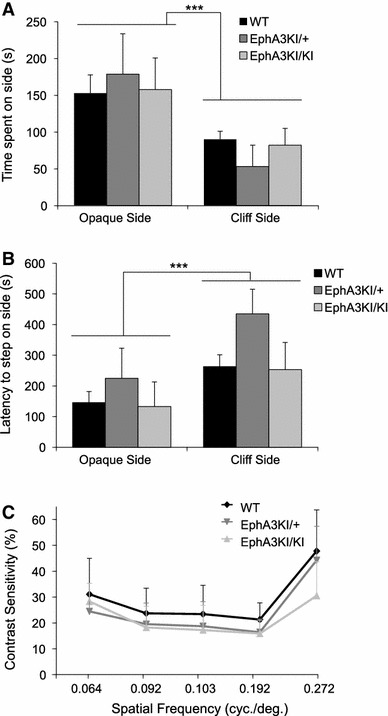
Visual acuity in Isl2-EphA3 knock-in mice. **a** In the visual cliff test, WT, EphA3KI/+ and EphA3KI/KI mice spent significantly more time on the opaque side compared to the cliff side. The three groups of mice did not differ in terms of mean time (s) spent on the opaque side and cliff side during the 10 min session. **b** The latency to step down toward the opaque side was significantly lowered compared to the cliff side, but similar in all genotypes. **c** In the OKR test, the average contrast sensitivity (threshold contrast as  %, y axis) for spatial frequencies ranging from 0.064 to 0.272 cycles/degree (*x* axis) varies similarly in the three groups of mice. ***p < 0.0001

We first asked whether the modified collicular retinotopy affects visual acuity using the visual cliff test, which measures visual depth perception in rodents. Mice from all three experimental groups spent significantly more time on the opaque side compared to the cliff side (side: F1,18 = 10.15, *p* = 0.005; Fig. 2a) and stepped earlier onto the opaque side than onto the cliff side (side: F1,18 = 16.61, *p* < 0.001; Fig. 2b) indicating normal visual perception. There was no significant difference between genotypes for the latency to step down and the time spent on either the checkered side or the cliff side (no genotype effect or genotype × side interaction). We next tested visual acuity by stimulating and measuring the optokinetic reflex (OKR). This reflex mediates compensatory head motions elicited by moving full-field visual stimuli, to maintain a constant image on the retina. Mice from all three genotypes showed similar threshold values for the minimum contrast that triggers an OKR at spatial frequencies ranging from 0.064 to 0.272 cycles/degree (Fig. 2c). Together, these results indicated normal visual acuity in EphA3^KI/KI^ and EphA3^KI/+^ mice.

### General locomotor activity, sensory motor coordination and circadian rhythm

**Fig. 3:**
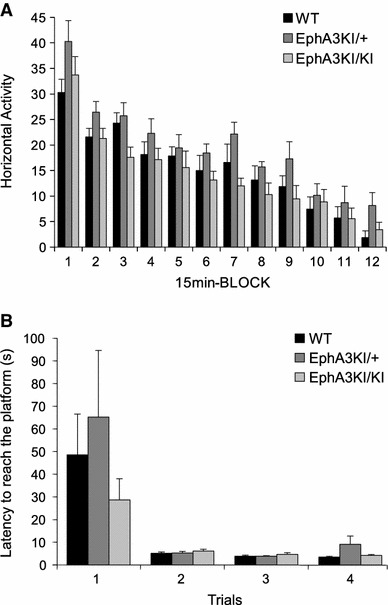
Locomotor activity and sensorimotor coordination in Isl2-EphA3 knock-in mice. **a** During the 3-h habituation phase, EphA3KI/KI and EphA3KI/+ mice did not differ from their WT littermates in terms of exploration of a new environment (expressed as mean horizontal activity per 15-min block). **b** EphA3KI/KI mice did not differ from their WT littermates in terms of mean time per trial to reach the platform over 4 trials of the beam test. In all three genotypes, this parameter decreased significantly over consecutive trials

**Fig. 4:**
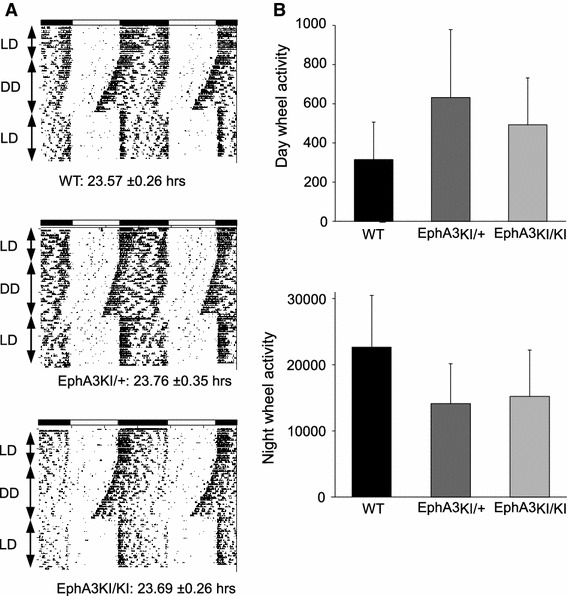
Circadian activity in Isl2-EphA3 knock-in mice. All three groups of mice showed similar endogenous periods after a 15 days of light–dark (LD) cycle followed by 10 days of constant darkness (DD) (**a**) and similar diurnal and nocturnal wheel running activity (**b**)

We next tested locomotor activity using horizontal cage activity and wheel running. Mice of each experimental group showed a similar decrease in locomotor activity over the course of a 3-h session corresponding to habituation to the new cage (15-min block: F11,198 = 55.17, *p* < 0.0001; Fig. 3a) and no significant effect of the genotype was observed in total wheel running activity, all three genotypes showing normal rhythmic activity (Fig. 4b, Online resource 2). The key role of the SC in the integration of sensorimotor modalities led us to test sensorimotor coordination. All three genotypes underwent the beam walking test and showed similar latencies to leave the start segment (genotype x trial: F6,54 = 0.65, *p* > 0.10, not shown) and to reach the platform, which decreased significantly during subsequent trials (trial: F3,54 = 16.48, *p* < 0.0001; Fig. 3b). Sensorimotor coordination and latency to leave the start segment were similar among genotypes. Moreover, we tested whether the running activity of knock-in mice follows light-entrained and endogenous circadian patterns. All three genotypes showed similar running activity in 12 h light–dark and dark–dark cycles with similar endogenous period (WT: 23.57 ± 0.26 h, EphA3^KI/+^: 23.76 ± 0.35 h and EphA3^KI/KI^: 23.69 ± 0.26 h; Fig. 4). Together these results indicate normal locomotor activity, sensory motor processing and circadian activity in EphA3^KI/KI^ and EphA3^KI/+^ animals.

### Visuo-spatial orientation and memory

**Fig. 5:**
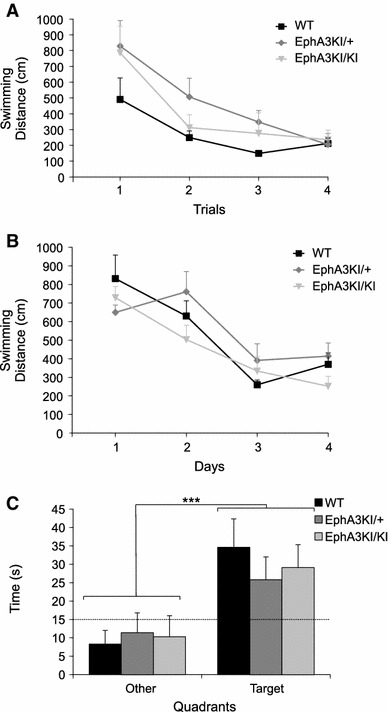
Visuo-spatial orientation, spatial navigation, learning and memory in Isl2-EphA3 knock-in mice. **a** In the visible platform test of the Morris water maze paradigm, all three groups of mice required similar mean swimming distances per trial to reach the visible platform and showed a similar decrease in the swimming distance over consecutive trials. **b** During the 4-day-long training period in the hidden platform test of the Morris water maze paradigm, Isl2-EphA3 knock-in mice and their WT littermates required similar swimming distances to reach the platform and showed a similar decrease over consecutive trials. **c** In the 60-s probe test without platform, mice spent significantly more time in the target quadrant compared to the mean time in other quadrants regardless of their genotype. ***p < 0.0001

We then tested vision and motor skills using the Morris water maze visible platform test, where mice must locate a cue at close range, and swim toward it. After 2 days of habituation, mice were tested for their performance in reaching a visible platform. Swim speed and distance were measured in four trials. Swim speed remained stable and similar for all groups. Swimming distance was similarly reduced among all groups over the four consecutive trials (trial: F3,54 = 16.07, *p* < 0.0001). No significant difference was observed among genotypes or genotype × trial interactions (Fig. 5a). Next we used a variant of the Morris water maze test where the platform is hidden to evaluate visuo-spatial learning and memory. Here, mice must find the hidden platform based on distant visual cues outside the pool. Over the course of the four training days, mice of all three genotypes showed similar swim speeds and learned the position of the hidden platform equally well (day: F3,54 = 20.67, *p* < 0.0001; Fig. 5b). No difference was observed between genotypes, suggesting that EphA3^KI/+^ and EphA3^KI/KI^ animals are able to learn a task requiring visuo-spatial orientation abilities. In a probe test performed 24 h later, all mice showed a clear bias toward the target quadrant where they spent significantly more time than the 15-s chance level (WT: *t*_6_ = 6.68, *p* = 0.0005, EphA3^KI/+^: *t*_6_ = 4.62, *p* = 0.004; EphA3^KI/KI^: *t*_6_ = 6.01, *p* = 0.001; Fig. 5c). Taken together, these results indicated normal visuo-spatial orientation, preserved motivation to reach a visible and hidden platform and intact spatial learning and memory in EphA3^KI/KI^ and EphA3^KI/+^ mice.

### Anxiety, response inhibition

**Fig. 6:**
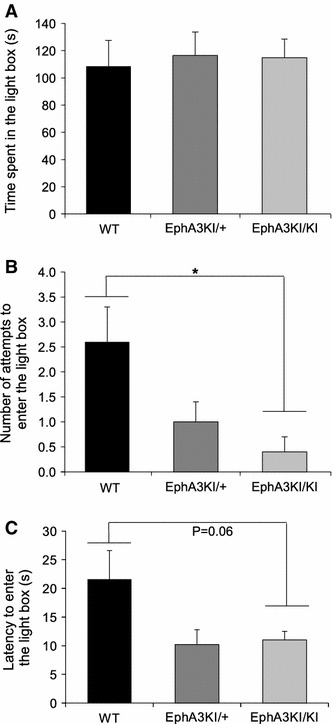
Anxiety-related behavior in Isl2-EpA3 knock-in mice. **a** In the light/dark box test, Isl2-EphA3 knock-in mice spent the same amount of time (s) in the light box as their WT littermates. **b** EphA3KI/KI animals showed a significant decrease in the number of attempts to enter the light box compared to the WT littermates. **c** EphA3KI/KI and EphA3KI/+ animals showed a tendency to a decreased latency (s) to enter the light box compared to their WT littermates. *p < 0.05

As the behavioral output in several tasks (e.g., visual cliff, Go/No-Go and Morris water maze) can be modulated by levels of anxiety, they were determined in the Isl2-EphA3 knock-in mice using the light/dark box test (Crawley 2007). This conflict test evaluates anxiety based on the tendency of a mouse to explore a novel environment against the aversive effect of a brightly lit open field (the light box). We measured both the time spent in the light box (aversive environment) and the number of attempts to enter this box (defined as an incomplete body entrance). Animals from the three genotypes spent a similar amount of time in the aversive environment (the light box) indicating comparable levels of anxiety (Fig. 6a). In support of that, habituation times in a novel activity cage and latency to leave the start segment in the beam walking test, presented above, did not differ between the three genotypes further suggesting that the Isl2-EphA3KI animals exhibit normal levels of anxiety. Surprisingly, EphA3^KI/KI^ and EphA3^KI/+^ mice made significantly fewer attempts to enter the light box (incomplete body entrances) compared to their WT littermates (attempts: F2,21 = 4.24, *p* < 0.05, NK post hoc: *p* < 0.05; Fig. 6b). In other words, EphA3^KI/KI^ and EphA3^KI/+^ mice were less hesitant and entered the light box more readily suggesting that they fail to refrain from exploring an aversive environment. In addition, EphA3^KI/KI^ and EphA3^KI/+^ mice showed a decreased latency for complete body entrance into the light box compared to WT littermates (latency: F2,21 = 3.24, *p* = 0.06; Fig. 6c). This provides further evidence that they did not hesitate to enter an aversive environment. However, EphA3^KI/KI^ mice showed no increase in time spent in the light box and no impairment in the visual cliff test, optokinetic reflex and both versions of the water maze in which performance depends on intact visual abilities (Yassine et al. 2013). Alternatively, reduced hesitation to enter the light box could be related to a diminished response inhibition, a key feature of impulsivity (Chamberlain and Sahakian 2007).

**Fig. 7:**
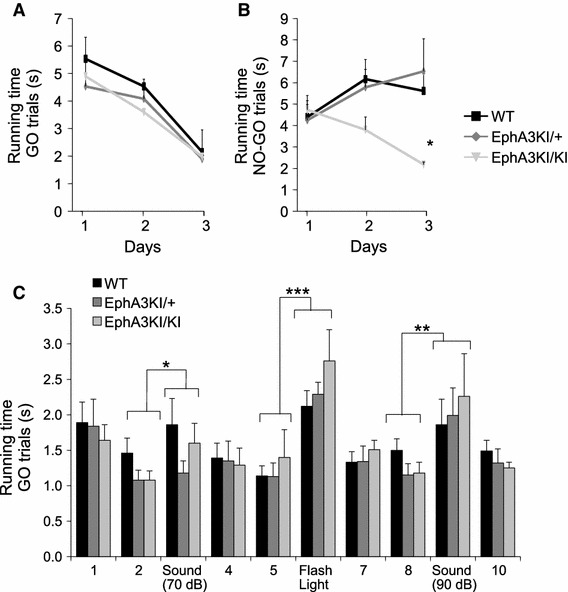
Go/No-Go performance in Isl2-EpA3 knock-in mice. **a** Over the three sessions, WT, EphA3KI/+ and EphA3KI/KI mice reduced their mean running time per trial in the reinforced Go trials. **b** Over the three sessions, WT and EphA3KI/+ mice show stable mean running time in the non-reinforced No-Go trials, as opposed to EphA3KI/KI littermates, which also reduced their running times in No-Go trials NK *p < 0.05. **c** Auditory (70, 90 dB tone) and visual (flash light) distractors led to significant increases in the running times in Go trials of all three genotypes. Note that EphA3KI/KI mice appeared slightly more sensitive to a visual distractor than their littermates. *p < 0.05; **p = 0.01; ***p < 0.0001

To confirm defects in response inhibition of knock-in mice, we performed a Go/No-Go task. Go/No-Go paradigms are based on a cue discrimination conditioning and are commonly used to assess attention and response inhibition, but also learning and memory functions in humans and mice (Meziane et al. 1993; Aron and Poldrack 2005; Gubner et al. 2010; Loos et al. 2010). This test required food restriction, during which the mice were kept at 85 % of their weight to ensure motivation for food reward. Mice of all three genotypes showed similar weight loss and motivation for food during food restriction (not shown) (Meziane et al. 1993). In our version of the task, mice were conditioned to run successively down two runaways differing in colors, one color runaway being always baited with food (Go trail) and the other never baited (No-Go trial). Both EphA3^KI/+^ and WT littermates progressively learned to discriminate between the reinforced (Go trials) and non-reinforced (No-Go trials) runways as indicated by a significant decrease in running time on Go trials and stable running times on No-Go trials (Go trials: F2,34 = 18.9, *p* < 0.0001; Fig. 7a, b) as usually observed in this task (Meziane et al. 1993). This suggested normal learning, motivation and response inhibition in EphA3^KI/+^ and WT mice. Running duration of EphA3^KI/KI^ animals decreased similarly than WT and EphA3^KI/+^ littermates on Go trials. Surprisingly, and in contrast to WT and EphA3^KI/+^, EphA3^KI/KI^ running times also significantly decreased on No-Go trials (No-Go trails: F4,34 = 4.03, *p* < 0.01, NK *p* < 0.05; Fig. 7a, b) indicating their failure to refrain themselves from running in the non-reinforced runway on No-Go trials. Preserved performances of the EphA3^KI/KI^ animals on Go trials suggested intact motivation for food and efficient learning. A discrimination learning deficit in these mice is unlikely since amnesic treatments are known to affect essentially Go running times (Meziane et al. 1993, 1998). In addition, their performance in the visible and hidden versions of the Morris water maze as well as in the visual cliff test and optokinetic reflex suggests that their visual acuity and visuo-spatial memory are comparable to those of WT and EphA3^KI/+^ littermates. Taken together, these results further support the hypothesis of a defective response inhibition in the EphA3^KI/KI^ animals.

In principle, this defective behavior could be caused by impaired attention or increased distraction (Barkley 2004). To test this possibility, we repeated the reinforced Go task, but added visual (flashing light) and auditory (tone) distractors. Mice of all genotypes showed significantly increased running times by reducing their speed in trials with tones (70 dB tone: F1,18 = 5.48, *p* < 0.05; 90 dB tone: F1,18 = 9.18, *p* < 0.01; Fig. 7c) and flash lights (F1,18 = 92.06, *p* < 0.0001; Fig. 7c) compared to non-distracted trials. Notably, all EphA3^KI/KI^ mice increased their running times when exposed to a flashing light, (one mouse stopped to look toward the origin of the stimulus) although the difference between EphA3^KI/KI^ and WT littermates did not reach statistical significance (Flash latency: F2,18 = 1.17, *p* = 0.33; Fig. 7c). These data indicate that a flashing light and loud tones are effective distractors during the Go task.

### Analysis of regional monoamine levels

**Fig. 8:**
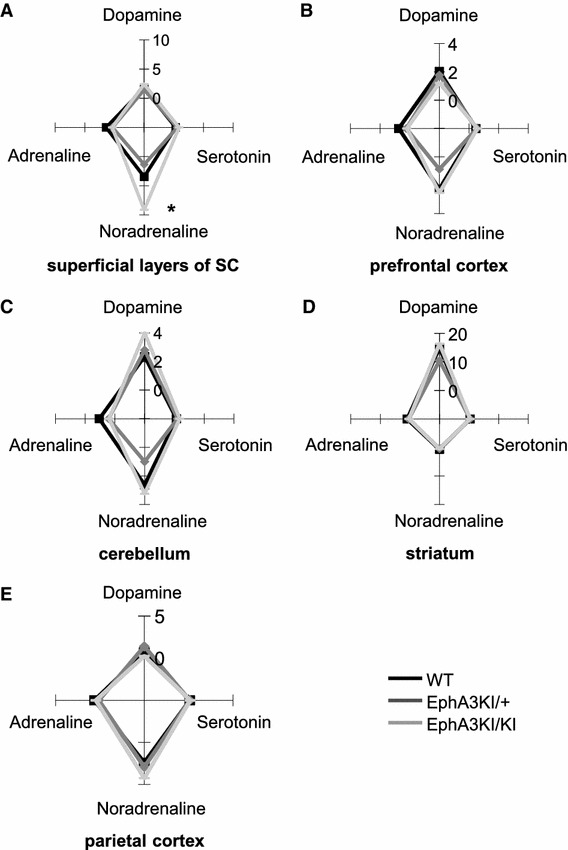
Monoamine concentrations in selected brain regions of Isl2-EphA3 knock-in mice. Radar-plot representation of total dopamine, adrenaline, noradrenaline and serotonin content (median values, ng/mg of proteins) in the **a** superficial layers of the SC, **b** prefrontal cortex, **c** cerebellum, **d** striatum and **e** parietal cortex. The noradrenaline content was significantly increased in superficial SC layers of EphA3KI/KI compared to EphA3KI/+ and WT littermates. *p < 0.05 KW test. *SC* superior colliculus

**Fig. 9:**
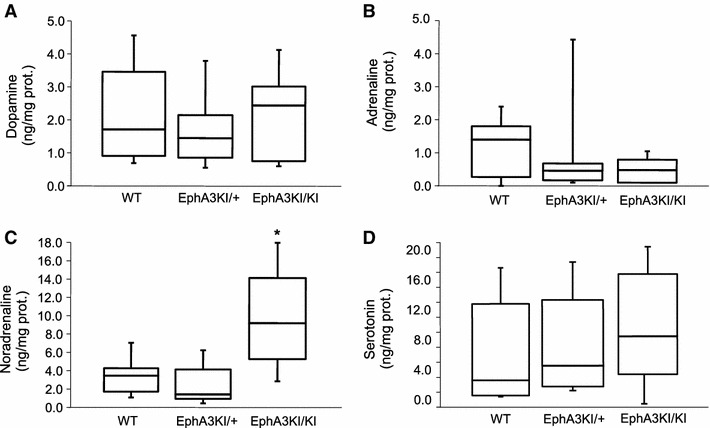
Monoamine content in the SC of Isl2-EphA3 knock-in mice. Boxplot representation (min, q1, median, q3, max) of total **a** dopamine, **b** adrenaline, **c** noradrenaline and **d** serotonin content (in ng/mg of proteins) in the superficial layers of the superior colliculus (SC) showing significant increase in noradrenaline in EphA3KI/KI animals compared to EphA3KI/+ and WT littermates. *p < 0.05 KW test

The observed defective response inhibition in EphA3^KI/KI^ mice, corresponding to an ADHD phenotypic feature, could be induced by abnormal catecholamine levels (van der Kooij and Glennon 2007; Sontag et al. 2010). To test this possibility, we determined levels of monoamine neurotransmitters in distinct areas of the mouse brain, namely the superficial layers of the superior colliculus (SC), the prefrontal cortex, the striatum, the parietal cortex and the cerebellum, all involved in attentional processes and motor control (Himelstein et al. 2000; Aron and Poldrack 2005; Biederman and Faraone 2005; Overton 2008). Levels of dopamine, adrenaline and serotonin were not significantly different between genotypes in the five structures studied (Fig. 8; Online resource 2). In contrast, the levels of noradrenaline were significantly increased in the superficial layers of the SC of EphA3^KI/KI^ compared to their EphA3^KI/+^ and WT littermates (KW test *p* < 0.05; Figs. 8a, 9). The increase in noradrenaline in the superficial layers of the SC prompted us to examine the expression of receptors, transporters and enzymes that are involved in monoaminergic metabolism and associated with attention-deficit diseases (Himelstein et al. 2000; Biederman and Faraone 2005). All three genotypes showed similar expression of transporters, metabolic enzymes and downstream receptors of dopamine, noradrenaline, adrenaline and serotonin in the superficial layers of the SC and in other brain regions (Online resource 2).

## Discussion

Our study provides first evidence for specific behavioral and molecular changes in mice with genetically altered retinotopy in the superior colliculus and consequently enhanced visual inputs. In the Go/No-Go task, EphA3^KI/KI^ mice performed normally on Go trials by increasing their running speed, but they were completely unable to inhibit their running response on No-Go trials.

In the light/dark box test, EphA3^KI/KI^ mice entered the aversive light box more readily than control mice. Altogether, our behavioral tests revealed that EphA3^KI/KI^ mice exhibit defective response inhibition, a form of impulsivity. The observation that heterozygous EphA3^KI/+^ mice behave like WT littermates in the Go/No-Go task suggests that a partial duplication of the retino-collicular map (Brown et al. 2000) is not sufficient to trigger defective response inhibition. The observed behavioral changes were remarkably specific, as all other paradigms tested, namely vision, visuo-spatial orientation, sensorimotor function, motivation, learning and memory as well as exploratory behavior and anxiety were similar in WT, EphA3^KI/+^ and EphA3^KI/KI^ mice. Defective response inhibition could be the consequence of enhanced levels of noradrenaline that we detected in the superficial layers of the SC of EphA3^KI/KI^ mice. Enhanced noradrenaline levels in the SC could alter the behavior of the EphA3^KI/KI^ mice by modulating the signal-to-noise ratio in this structure (Mooney et al. 1990; Tan et al. 1999) and thereby changing its level of activation (Dommett et al. 2009). In hamsters, in vivo and in vitro studies demonstrated a suppression of collicular neuron response upon noradrenaline application (Mooney et al. 1990; Tan et al. 1999). In rats, Sato and Kayama reported that iontophoretically applied noradrenaline exerts an excitatory action, indicating an increase of the signal-to-noise ratio, in accordance with our hypothesis (Sato and Kayama 1983). Whether noradrenaline increases or decreases the signal-to-noise ratio in the superficial layers of the SC is still debated. However, it clearly affects the processing of salient stimuli in a context-specific manner (Sato and Kayama 1983; Mooney et al. 1990; Tan et al. 1999).

The increase in noradrenaline was specific to the superficial layers of the SC, where the retinotopy is duplicated. Moreover, the increase only concerned noradrenaline, whereas other monoamines including dopamine, serotonin and adrenaline showed similar concentrations for all genotypes and brain regions. The increase in noradrenaline was not accompanied by changes in transcript levels of genes involved in monoamine metabolism. Therefore, we hypothesize that the increase of noradrenaline in the superficial layers of the SC may be the consequence of the duplication of the RGCs projections, which are functional, as shown by optical intrinsic imaging (Triplett et al. 2009). Previous studies revealed that RGC axons release noradrenaline upon activation (Osborne and Patel 1985). Alternatively, the increase may come from a duplication of projections from the *locus coeruleus* (LC), the major source of noradrenaline in the brain, to the superficial layers of the SC (Takemoto et al. 1978; Fritschy et al. 1990). Whether LC projections to the SC are duplicated is unknown as the mapping of the LC to the SC is hindered by the small size and specific sub-nuclei organization of the LC. However, it appears possible given that cortico-collicular projections are also duplicated in the EphA3^KI/KI^ animals although projecting V1 neurons do not express ectopic EphA3 (Triplett et al. 2009). RGCs project to different brains areas, including lateral geniculate nucleus (LGN) and non-image forming structures such as the suprachiasmatic nucleus (SCN), the medial tegmental nucleus (MTN) or the olivary pretectal nucleus (OPN). Triplett and colleagues show no targeting defects in the LGN of Isl2-EphA3 animals (Triplett et al. 2009). The same group recently demonstrated that among 1 % of RGCs projecting to the SCN (the intrinsically photoreceptive RGCs—ipRGCs), 3 % are Isl2-positive and that these SCN-targeting Isl2-positive RGCs only transiently innervate the SCN during the development (Triplett et al. 2014). MTN and OPN also show innervation by Isl2-positive RGCs at early postnatal stages which is pruned by P6 (Triplett et al. 2014). The behavioral and molecular changes in EphA3^KI/KI^ mice including defective response inhibition and noradrenaline enrichment in the superficial layers of the SC phenocopy some of the symptoms observed in ADHD patients, specifically the adult and predominantly inattentive-type (Barkley 1997; Aron and Poldrack 2005; Biederman and Faraone 2005; Bekker et al. 2005; Fisher et al. 2011; American Psychiatric Association 2013). These symptoms are also main features of Autism Spectrum Disorder (ASD) (Murray 2010). Our findings support the hypothesis that adult ADHD patients present collicular hyperstimulation leading to the appearance of impulsivity and attentional impairments (Overton 2008; Miller 2009; Dommett et al. 2009). Moreover, they are in line with the idea that dysregulation of the central noradrenergic systems contributes to the pathophysiology of ADHD (Biederman and Spencer 1999). Currently, progress on the etiology, diagnosis and treatment of ADHD is hindered by the limited number of experimental models. Most of the available rodent models are based on impaired monoaminergic transmission (van der Kooij and Glennon 2007; Sontag et al. 2010) and present some of the phenotypic features of ADHD patients. Our findings suggest that EphA3^KI/KI^ animals may serve as a new model to study ADHD pathology and complement the limited arsenal of ADHD/ADD-related experimental approaches to understand and treat these neuropsychologic diseases.

## Electronic supplementary material

Below is the link to the electronic supplementary material.

Supplementary material 1 (DOC 49 kb)

Supplementary material 2 (DOC 88 kb)

## References

[CR1] American Psychiatric Association (2013) Diagnostic and statistical manual of mental disorders, 5th edn. doi: 10.1176/appi.books.9780890423349

[CR2] Aron AR, Poldrack RA (2005). The cognitive neuroscience of response inhibition: relevance for genetic research in attention-deficit/hyperactivity disorder. Biol Psychiatry.

[CR3] Barkley RA (1997). Behavioral inhibition, sustained attention, and executive functions: constructing a unifying theory of ADHD. Psychol Bull.

[CR4] Barkley RA (2004). Driving impairments in teens and adults with attention-deficit/hyperactivity disorder. Psychiatr Clin North Am.

[CR5] Bekker EM, Overtoom CCE, Kooij JJS (2005). Disentangling deficits in adults with attention-deficit/hyperactivity disorder. Arch Gen Psychiatry.

[CR6] Bevins N, Lemke G, Reber M (2011). Genetic dissection of EphA receptor signaling dynamics during retinotopic mapping. J Neurosci.

[CR7] Biederman J, Faraone SV (2005). Attention-deficit hyperactivity disorder. Lancet.

[CR8] Biederman J, Spencer T (1999). Attention-deficit/hyperactivity disorder (ADHD) as a noradrenergic disorder. Biol Psychiatry.

[CR9] Binns KE (1999). The synaptic pharmacology underlying sensory processing in the superior colliculus. Prog Neurobiol.

[CR10] Boeuf J, Trigo JM, Moreau P-H (2009). Attenuated behavioural responses to acute and chronic cocaine in GASP-1-deficient mice. Eur J Neurosci.

[CR11] Brown A, Yates PA, Burrola P (2000). Topographic mapping from the retina to the midbrain is controlled by relative but not absolute levels of EphA receptor signaling. Cell.

[CR12] Chamberlain SR, Sahakian BJ (2007). The neuropsychiatry of impulsivity. Curr Opin Psychiatry.

[CR13] Crawley JN (2007). What’s Wrong With My Mouse: Behavioral Phenotyping of Transgenic and Knockout Mice.

[CR14] Diamond A (2005). Attention-deficit disorder (attention-deficit/hyperactivity disorder without hyperactivity): a neurobiologically and behaviorally distinct disorder from attention-deficit/hyperactivity disorder (with hyperactivity). Dev Psychopathol.

[CR15] Dommett EJ, Overton PG, Greenfield SA (2009). Drug therapies for attentional disorders alter the signal-to-noise ratio in the superior colliculus. Neuroscience.

[CR16] Dottori M, Hartley L, Galea M (1998). EphA4 (Sek1) receptor tyrosine kinase is required for the development of the corticospinal tract. Proc Natl Acad Sci USA.

[CR17] Douglas RM, Alam NM, Silver BD (2005). Independent visual threshold measurements in the two eyes of freely moving rats and mice using a virtual-reality optokinetic system. Vis Neurosci.

[CR18] Feldheim DA (2004). Loss-of-function analysis of EphA receptors in retinotectal mapping. J Neurosci.

[CR19] Feldheim DA, Kim YI, Bergemann AD (2000). Genetic analysis of ephrin-A2 and ephrin-A5 shows their requirement in multiple aspects of retinocollicular mapping. Neuron.

[CR20] Fisher T, Aharon-Peretz J, Pratt H (2011). Dis-regulation of response inhibition in adult attention deficit hyperactivity disorder (ADHD): an ERP study. Clin Neurophysiol.

[CR21] Fritschy JM, Geffard M, Grzanna R (1990). The response of noradrenergic axons to systemically administered DSP-4 in the rat: an immunohistochemical study using antibodies to noradrenaline and dopamine-beta-hydroxylase. J Chem Neuroanat.

[CR22] Fuentes LJ (2001). Selective attention deficit in schizophrenia. Rev Neurol.

[CR23] Gandhi NJ, Katnani HA (2011). Motor functions of the superior colliculus. Annu Rev Neurosci.

[CR24] Gibson EJ, Walk RD (1960). The “visual cliff”. Sci Am.

[CR25] Gubner NR, Wilhelm CJ, Phillips TJ, Mitchell SH (2010). Strain differences in behavioral inhibition in a Go/No-go task demonstrated using 15 inbred mouse strains. Alcohol Clin Exp Res.

[CR26] Himelstein J, Newcorn JH, Halperin JM (2000). The neurobiology of attention-deficit hyperactivity disorder. Front Biosci.

[CR27] Holmes NP, Spence C (2005). Multisensory integration: space, time and superadditivity. Curr Biol CB.

[CR28] Huberman AD, Niell CM (2011). What can mice tell us about how vision works?. Trends Neurosci.

[CR29] King AJ, Schnupp JW, Thompson ID (1998). Signals from the superficial layers of the superior colliculus enable the development of the auditory space map in the deeper layers. J Neurosci Off J Soc Neurosci.

[CR30] Kleinhans NM, Richards T, Johnson LC (2011). fMRI evidence of neural abnormalities in the subcortical face processing system in ASD. NeuroImage.

[CR31] Lemke G, Reber M (2005). Retinotectal mapping: new insights from molecular genetics. Annu Rev Cell Dev Biol.

[CR32] Loos M, Staal J, Schoffelmeer ANM (2010). Inhibitory control and response latency differences between C57BL/6 J and DBA/2 J mice in a Go/No-Go and 5-choice serial reaction time task and strain-specific responsivity to amphetamine. Behav Brain Res.

[CR33] May PJ (2006). The mammalian superior colliculus: laminar structure and connections. Prog. Brain Res.

[CR34] Mendoza J, Pévet P, Challet E (2008). High-fat feeding alters the clock synchronization to light. J Physiol.

[CR35] Meredith MA, Stein BE (1985). Descending efferents from the superior colliculus relay integrated multisensory information. Science.

[CR36] Meziane H, Devigne C, Tramu G, Soumireu-Mourat B (1993). Effects of anti-CCK-8 antiserum on acquisition and retrieval by mice in an appetitive task. Peptides.

[CR37] Meziane H, Dodart JC, Mathis C (1998). Memory-enhancing effects of secreted forms of the beta-amyloid precursor protein in normal and amnestic mice. Proc Natl Acad Sci USA.

[CR38] Miller L (2009). Perspectives on sensory processing disorder: a call for translational research. Front Integr Neurosci.

[CR39] Mooney RD, Bennett-Clarke C, Chiaia NL (1990). Organization and actions of the noradrenergic input to the hamster’s superior colliculus. J Comp Neurol.

[CR40] Moreau P-H, Cosquer B, Jeltsch H (2008). Neuroanatomical and behavioral effects of a novel version of the cholinergic immunotoxin mu p75-saporin in mice. Hippocampus.

[CR41] Murray MJ (2010). Attention-deficit/hyperactivity disorder in the context of autism spectrum disorders. Curr Psychiatry Rep.

[CR42] Osborne NN, Patel S (1985). The presence of dopamine-?-hydroxylase-like enzyme in the vertebrate retina. Neurochem Int.

[CR43] Overton PG (2008). Collicular dysfunction in attention deficit hyperactivity disorder. Med Hypotheses.

[CR44] Reber M, Burrola P, Lemke G (2004). A relative signalling model for the formation of a topographic neural map. Nature.

[CR45] Ross KC, Coleman JR (2000). Developmental and genetic audiogenic seizure models: behavior and biological substrates. Neurosci Biobehav Rev.

[CR46] Sato H, Kayama Y (1983). Effects of noradrenaline applied iontophoretically on rat superior collicular neurons. Brain Res Bull.

[CR47] Sontag TA, Tucha O, Walitza S, Lange KW (2010). Animal models of attention deficit/hyperactivity disorder (ADHD): a critical review. ADHD Atten Deficit Hyperact Disord.

[CR48] Sperry RW (1963). Chemoaffinity in the orderly growth of nerve fiber patterns and connections. Proc Natl Acad Sci USA.

[CR49] Stein BE (1984). Development of the superior colliculus. Annu Rev Neurosci.

[CR50] Stein BE, Stanford TR, Rowland BA (2009). The neural basis of multisensory integration in the midbrain: its organization and maturation. Hear Res.

[CR51] Takemoto I, Sasa M, Takaori S (1978). Role of the locus coeruleus in transmission onto anterior colliculus neurons. Brain Res.

[CR52] Tan H, Mooney RD, Rhoades RW (1999). Effects of norepinephrine upon superficial layer neurons in the superior colliculus of the hamster: in vitro studies. Vis Neurosci.

[CR53] Thaler JP, Koo SJ, Kania A (2004). A postmitotic role for Isl-class LIM homeodomain proteins in the assignment of visceral spinal motor neuron identity. Neuron.

[CR54] Triplett JW, Owens MT, Yamada J (2009). Retinal input instructs alignment of visual topographic maps. Cell.

[CR55] Triplett JW, Phan A, Yamada J, Feldheim DA (2012). Alignment of multimodal sensory input in the superior colliculus through a gradient-matching mechanism. J Neurosci.

[CR56] Triplett JW, Wei W, Gonzalez C (2014). Dendritic and axonal targeting patterns of a genetically-specified class of retinal ganglion cells that participate in image-forming circuits. Neural Dev.

[CR57] Van der Kooij MA, Glennon JC (2007). Animal models concerning the role of dopamine in attention-deficit hyperactivity disorder. Neurosci Biobehav Rev.

[CR58] Wallace MT, Meredith MA, Stein BE (1993). Converging influences from visual, auditory, and somatosensory cortices onto output neurons of the superior colliculus. J Neurophysiol.

[CR59] Yassine N, Lazaris A, Dorner-Ciossek C (2013). Detecting spatial memory deficits beyond blindness in tg2576 Alzheimer mice. Neurobiol Aging.

