# Supplementary material for: Defective response inhibition and collicular noradrenaline enrichment in mice with duplicated retinotopic map in the superior colliculus
Source: Brain Struct Funct. 2014 Mar 20;220(3):1573–84. doi: 10.1007/s00429-014-0745-5 (PMC4409641; doi:10.1007/s00429-014-0745-5)
Supplement: Supplementary file 1 — Supplementary material 1 (DOC 49 kb) [file 429_2014_745_MOESM1_ESM.doc]

**Online Resource 1:**

**Supplemental Materials and methods**

**Behavioral testing of the first cohort**

A first cohort of four to seven-month old male littermates of all three genotypes (n = 6-9) was first tested in the light/dark box test (Boeuf et al. 2009) (ITI 5 days) as this test is highly sensitive to previous handling and then in the Go/No-Go task (Meziane et al, 1993) (ITI 5 days) which is a key task in our study. Other tasks were not performed afterward to avoid any interference with the prolonged food restriction period.

***Light/Dark box test***

To assess anxiety, which may affect behavioral outputssuch as running times in the Go/No-Go task, we performed single 5-min.light/dark box test on naïve males (Boeuf et al. 2009). The task was performed between ZT3 and ZT9 to match as much as possible with the period of testing in the Go/No-Go task. The apparatus consisted of two equal-sized PVC boxes (18.5 x 18.5 x 15 cm), one opaque and one transparent, connected by an opaque PVC tunnel (5 cm Ø, 5.5 cm long). A 100 W lamp was positioned 10 cm above the transparent box. The mouse was initially placed in the dark box and the number of attempts (incomplete body entrance) until the first complete light box entrance, the latency of this complete entrance and the total time spent in the light box were recorded during five minutes.

***Go/No-Go task: impulsivity***

Go/No-Go paradigms are based on a dual cue-dependent response expression/inhibition and are widely used to characterize response inhibition defects in patients with attention-deficit disorder (Meziane et al. 1993; Loos et al. 2010). To evaluate response inhibition in our animal model, the above mentioned cohort was tested in the visual discrimination Go/No-Go task. Males were tested between ZT2 and ZT10.The apparatus consisted of two side-by-side Plexiglas runways (68 cm long x 6.5 cm wide x 20 cm high walls) one black and one white. Each runway had a start box with a guillotine door and a goal box with a food well, but only one of the runways was associated with food reward (reinforced) for an individual mouse (pseudo-random attribution). Before training, the mice were habituated to the apparatus (5 min/runway), then progressively food-deprived over four days, while being familiarized with the reward pellets (durum wheat noodles, 17.3 ± 0.3 mg each). After initial food deprivation the mice were maintained at 80 – 85 % of their *ad libitum* body weight throughout the course of the experiment to ensure motivation for food reward. The experiments were carried out over five daily sessions with each session consisting of 12 consecutive trials, randomly organized in six individually reinforced (R) Go trials and six individually non-reinforced (NR) No-Go trials. The sequence of testing was R-NR-R-NR-NR-R-NR-R-R-NR-R-NR on the first day, R-NR-NR-R-R-NR-NR-R-R-R-NR-NR on the second day, and R-NR-R-NR-NR-R-NR-NR-NR-R-R-R on the last day. For each trial, the mouse was released from the start box of one runway and the latency to reach the goal box at the end of this runway (running time) was recorded (always less than 15 s.).

***Go task: distractibility***

The day following the Go/No-Go paradigm, distractibility of the mice was evaluated during a single session of ten Go trials using the reinforced runway. Each Go trial followed the same procedure as the one used in the Go/No-Go task except that auditive (tone) and visual (flash) distractors were delivered at the level of the start box as soon as the mice entered the runway. Distractors were delivered on trial 3 (tone: 2 KHz, 70 dB, 0.5 s), trial 6 (flash light, 1.2 ms) and trial 9 (tone: 2 KHz, 90 dB, 0.5 s). Thus, each mouse was exposed only once to a given distractor. The ANY-maze video-tracking system and the AMI-interface (Ugo Basile, Italy) were used to control the delivery of the distractors and to record the running times. The effect of the distractor was evaluated by comparing the running times on tone/flash trials to those of the preceding trial.

**Behavioral testing of the second cohort**

The second cohort of four to seven-month old males littermates (n = 7 per group) was dedicated to sensorimotor , learning and memory evaluations. They were first tested for circadian wheel-running activity (Mendoza et al. 2008) and general locomotor activity (Yassine et al. 2013) (ITI 15 days) followed by the Morris water maze paradigm (Moreau et al. 2008) (ITI 15 days), the beam walking test (Moreau et al. 2008) (ITI 3 days) and the visual cliff test (Gibson and Walk 1960) (ITI 21 days).

***Circadian phenotyping***

Circadian phenotyping was performed for 15 days in light-dark cycle (LD 12h/2h , 07.00-19.00h) and for 10 days in constant darkness (DD) to monitor circadian expression of wheel-running behaviour as described (Mendoza et al. 2008). The mice were housed in a temperature- and humidity- controlled room (22±1°C and 55 ± 10%, respectively) with free access to food and water. They were placed in individual plexiglas cages (Ehret mouse type 2: 26.5x20.5x14cm) equipped with a running-wheel (diameter 11cm) to measure locomotor activity. Wheel revolutions were recorded and stored in 5min-time bins by a computerized recording system (VitalView software, Mini Mitter Co., Sunriver, OR, USA). Daily rhythms of wheel-running activity were analyzed and plotted as actograms using with ClockLab software (V 1.0, Actimetrics, Evanston, IL, USA). Daily activity was defined as the total number of wheel revolutions performed per day for each mouse over the last 10 days of LD and DD. In constant darkness, calculation of the endogenous period (Tau) was performed with χ^2^ periodogram (ClockLab).

***General locomotor activity***

Mice were tested in an actography device to evaluate locomotor activity as described (Boeuf et al. 2009; Yassine et al. 2013). Mice were individually placed in large transparent Makrolon cages (42 x 26 x 15 cm^3^) intersected by two infrared light beams, targeted on two photo-sensitive cells, 2.5 cm above the cage floor level and 28 cm apart. The horizontal cage crossings were counted in 15-minute bins during the first 3 hours and then in 1-hour bins over the next 69 hours.

***Morris water-maze test***

The apparatus and general procedures were described previously (Moreau et al. 2008). The circular pool (Ø: 140 cm, height: 40cm) filled with 20°C opacified water was located in an experimental room providing extra-maze visual cues (e.g. rack, water heater, pictures and overhead fluorescent white room lighting). Mice were released facing the edge of the tank in one of the four standard starting points, identified as north (N), east (E), south (S) and west (W), which divide the pool in four equal, virtual quadrants (SW, SE, NW and NE). The mice were first trained for four trials in a day with a water depth of 20 cm and a visible platform (Ø: 10 cm) protruding 0.5 cm above the water surface to provide an escape. For each trial, the location of the visible platform (middle of one quadrant: SW, SE, NE or NW) and the starting points (N, E, S, or W) were changed in a pseudo-random fashion. Training in the spatial memory version of the task, for which the platform was hidden by submersion, began three days later and lasted for 4 days. On each trial, the mouse had 60 seconds to find the submerged platform that was located in the middle of the SW quadrant. The pseudo-random order of the four possible starting points was changed from day to day. The latency and the distance to reach the hidden platform were recorded for each trial using a video-tracking system (Ethovision, Noldus, The Netherlands). On the following day, after removal of the platform, a 60-seconds probe trial was performed to evaluate whether the mice remembered the position of the hidden platform (strength and precision of spatial memory).

***Beam walking test***

The beam-walking test was used to evaluate sensorimotor skills and performed as described (Moreau et al. 2008). Each mouse was placed at the extremity of a 1 cm wide, 80 cm long beam maintained at a 90 cm height by a tripod. For four consecutive trials, the mouse had to walk on the beam to a small platform from where it could enter its home cage. A thick security pad was positioned under the testing device. The latency to leave the 10 cm long start segment of the beam and the total duration of the 80 cm trip to the platform were recorded.

***Visual cliff test***

Depth perception was measured using a modified version of the visual cliff apparatus (Gibson and Walk 1960). The apparatus consists of a 54 x 54 cm open field bordered by 40 cm high walls. The floor of the open field is composed of a “safe zone” made of an opaque bottom (54 x 9 cm) and a “virtual cliff zone” made of transparent plexiglas, giving the impression of a cliff. The single 10-min trial started by placing the mouse on a 54 x 9 x 4 cm platform, which was located between the “safe zone” and the “virtual cliff zone”. Mice having normal vision are expected to step from the starting platform onto the “safe zone”. For each trial, the latency to step down on each side and the time spent on the platform and in each zone were recorded using a video-recording system (ANY-maze, Ugo Basile, Italy).

**Behavioral testing of the third cohort**

***Optokinetic reflex - visual contrast and spatial frequencies perception***

For these experiments, mice were bred and maintained at the mouse facility of the Salk Institute (San Diego, CA, USA). All procedures used in these experiments were reviewed and approved by the Institutional Animal Care and Use Committee (IACUC) of the Salk Institute. Animals were cared for and used in accordance with guidelines of the U.S. Public Health Service Policy on Humane Care and Use of Laboratory Animals and of the NIH Guide for the Care and Use of Laboratory Animals and following institutional Association for Assessment and Accreditation of Laboratory Animal Care-approved practices. Measurements of the optokinetic reflex in freely moving animals were performed as described (Douglas et al. 2005). Briefly, a freely moving mouse was located on the test platform surrounded by 4 computer monitors creating a virtual cylinder. Visual stimuli were composed of alternating vertical stripes projected on the monitors. The contrast, spatial frequency and speed of rotation of the visual stimuli were controlled by the experimenter. A camera positioned above the platform allowed for direct observation of the mouse interacting with its virtual environment. If the mouse moved its head in the direction of the rotation after 3 stimuli lasting 10 seconds each, it was counted as “tracking”. If the mouse did not move its head in the proper direction it was counted as “not tracking”. Threshold contrast values (contrast of the alternating stripes) for tracking at 20 degrees/second velocity and spatial frequencies of 0.064, 0.092, 0.103, 0.192, and 0.272 cycles/degree were obtained using a stepwise algorithm as described^6^. Contrast sensitivity, i.e. the ability to distinguish alternating stripes for a given contrast, was measured at a constant velocity of 20 degrees/second and at spatial frequencies between 0.064 and 0.272 cycle/degree. Threshold values for both the clockwise (CW) and counter-clockwise (CCW) direction were obtained for each animal and treated as independent measurements. The mean threshold values were compared between groups using a two-tailed t-test.

***RNA extraction and Semi-quantitative PCR***

Quantitative PCR was used to compare the transcriptional level of mRNA in different groups of mice. Total RNA was isolated by homogenization of samples in TRIzol reagent (Ambion life technology) followed by chloroform extraction. Further processing was performed according manufacturer’s instruction (PureLink RNA Mini Kit, Ambion, Life technologies). Relative transcript levels were quantified as described (Passemard et al. 2011) The following genes representing molecular pathways of monoamine signaling were analyzed: DAT (Dopamine transporter), 5HT1A (Serotonin receptor 1A), 5HT1B (Serotonin receptor 1B), MAO-A (Mono-amine oxidase A), PNMT (Phenylethanolamine N-methyl transferase), DBH (Dopamine beta hydroxylase), SNAP25 (Synaptosomal-associated protein 25), D1A (Dopamine receptor 1A), D2 (Dopamine receptor 2), TH (Tyrosine hydroxylase), Adra2A (Adrenoreceptor 2A), Adra2C (Adrenoreceptor 2C), NET (Noradrenalin transporter) (primer sequences available upon request).

Total RNA was isolated by homogenization of samples in TRIzol reagent (Ambion life technology) and separated by chloroform. Supernatant was transferred and processed with a commercial kit according to manufacturer’s instruction (PureLink RNA Mini Kit, Ambion, Life technologies). Genomic DNA was removed by digestion with RNase-free DNase I (Ambion). RNA quality and concentration was evaluated with Agilent 2100 Bioanalyzer. Relative quantification was performed as described (Passemard et al. 2011). Briefly, complementary DNA (cDNA) was generated from 250 ng of RNA using iScript Reverse Transcriptase kit. qPCR was performed with iQ SYBR Green Supermix (Bio-Rad) on an iQ5 Real Time PCR System (Bio-Rad). PCR cycling conditions were 95°C for 3 min followed by 49 cycles of 95°C for 20 sec, 62°C for 20 sec, 72°C for 20sec.

Relative quantification was performed with the standard curve method, which takes into account the efficiency of amplification. For each sample, concentration for the target gene and for two house keeping genes (hypoxanthine-guanine phosphoribosyltransferase - HPRT and glyceraldehyde 3-phosphate dehydrogenase - GAPDH) were computed according to a standard curve, consisting in serial dilutions of cDNA matched with the sample. A standard for the target gene was performed at each trial and used to obtain the relative expression when compared to HPRT and GAPDH expression.

**Monoamine extraction and quantification by HPLC**

Brain structures were dissected in PBS and homogenized in water and proteins were precipitated by perchloric acid (0.1N final) and ascorbic acid (2.5mM) was added to prevent monoamine oxidation. After centrifugation (16 kRCF, 15 min at 4°C), supernatants were recovered and stored at -20°C until further analysis. For quantification, samples were loaded into a reverse-phase HPLC column (Macherey-Nagel, Nucleosil RP100-5 C18 HD, 4x250mm) and fluorescence of eluted compounds was monitored at 340nm upon excitation at 270nm. Elution was carried out at a flow rate of 0.7 ml/min using a mobile phase A (5mM H_3_PO_4_, 5mM hexasulfonic acid) and B (70 % acetonitrile; v:v) with the following gradient : 1% of B/min from 0 to 32min followed by 8% of B/min from 32 to 44min. Standards of 500pmol of commercial dopamine, adrenaline and noradrenaline and 100pmol of serotonin (Sigma Aldrich) were used. Peaks of interest were identified according to retention time of these standards. Peak integration was performed by Unicorn software. A Bradford colorimetric assay (BioRad Protein Assay) was used to measure protein concentration and to normalize monoamine concentration.

**Supplemental References**

Boeuf J, Trigo JM, Moreau P-H, et al. (2009) Attenuated behavioural responses to acute and chronic cocaine in GASP-1-deficient mice. Eur J Neurosci 30:860–868. doi: 10.1111/j.1460-9568.2009.06865.x

Douglas RM, Alam NM, Silver BD, et al. (2005) Independent visual threshold measurements in the two eyes of freely moving rats and mice using a virtual-reality optokinetic system. Vis Neurosci 22:677–684. doi: 10.1017/S0952523805225166

Gibson EJ, Walk RD (1960) The “visual cliff.”Sci Am 202:64–71.

Loos M, Staal J, Schoffelmeer ANM, et al. (2010) Inhibitory control and response latency differences between C57BL/6J and DBA/2J mice in a Go/No-Go and 5-choice serial reaction time task and strain-specific responsivity to amphetamine. Behav Brain Res 214:216–224. doi: 10.1016/j.bbr.2010.05.027

Mendoza J, Pévet P, Challet E (2008) High-fat feeding alters the clock synchronization to light. J Physiol 586:5901–5910. doi: 10.1113/jphysiol.2008.159566

Meziane H, Devigne C, Tramu G, Soumireu-Mourat B (1993) Effects of anti-CCK-8 antiserum on acquisition and retrieval by mice in an appetitive task. Peptides 14:67–73.

Moreau P-H, Cosquer B, Jeltsch H, et al. (2008) Neuroanatomical and behavioral effects of a novel version of the cholinergic immunotoxin mu p75-saporin in mice. Hippocampus 18:610–622. doi: 10.1002/hipo.20422

Passemard S, El Ghouzzi V, Nasser H, et al. (2011) VIP blockade leads to microcephaly in mice via disruption of Mcph1-Chk1 signaling. J Clin Invest 121:3071–3087. doi: 10.1172/JCI43824

Yassine N, Lazaris A, Dorner-Ciossek C, et al. (2013) Detecting spatial memory deficits beyond blindness in tg2576 Alzheimer mice. Neurobiol Aging 34:716–730. doi: 10.1016/j.neurobiolaging.2012.06.016
