# Supplementary material for: Defective response inhibition and collicular noradrenaline enrichment in mice with duplicated retinotopic map in the superior colliculus
Source: Brain Struct Funct. 2014 Mar 20;220(3):1573–84. doi: 10.1007/s00429-014-0745-5 (PMC4409641; doi:10.1007/s00429-014-0745-5)
Supplement: Supplementary file 2 — Supplementary material 2 (DOC 88 kb) [file 429_2014_745_MOESM2_ESM.doc]

**Online Resource 2**

**Supplemental Figures and Tables**

**Supplemental Figure Legend**

Supplemental Figure S1: Night and day actigraphy over 72 hours in WT, EphA3KI/+ and EphA3KI/KI animals.

Supplemental Figure S2: Box-plot representation (min., q1, med., q3, max.) of (A) dopamine, (B) adrenaline, (C) noradrenaline and (D) serotonin total content (in ng/mg of proteins) in the prefrontal cortex of WT, EphA3KI/+ and EphA3KI/KI animals.

Supplemental Figure S3: Box-plot representation (min., q1, med., q3, max. of (A) dopamine, (B) adrenaline, (C) noradrenaline and (D) serotonin total content (in ng/mg of proteins) in the striatum of WT, EphA3KI/+ and EphA3KI/KI animals.

Supplemental Figure S4: Box-plot representation (min., q1, med., q3, max.) of (A) dopamine, (B) adrenaline, (C) noradrenaline and (D) serotonin total content (in ng/mg of proteins) in the parietal cortex of WT, EphA3KI/+ and EphA3KI/KI animals.

Supplemental Figure S5: Box-plot representation (min., q1, med., q3, max.) of (A) dopamine, (B) adrenaline, (C) noradrenaline and (D) serotonin total content (in ng/mg of proteins) in the cerebellum of WT, EphA3KI/+ and EphA3KI/KI animals.

Supplemental Table S1: qPCR semi-quantitation of monoaminergic transporters, receptors and enzymes and ADHD associated genes in the superficial layers of the superior colliculus of WT, EphA3KI/+ and EphA3KI/KI animals expressed as min, q1, med, q3, max. 5HT1A: serotonin receptor 1A, 5HT1B: serotonin receptor 1B, Adra2A: a-2A adrenergic receptor, Adra2C: a-2C adrenergic receptor, D1: dopaminergic receptor 1, D2: dopaminergic receptor 2, DAT: dopamine transporter, NET: noradrenalin transporter, TH: tyrosine hydroxylase, DBH: dopamine-b-hydroxylase, MAOA: monoamine oxidase A, PNMT: phenylethanolamine *N*-methyltransferase, SNAP25: synaptosomal-associated protein 25.

Supplemental Table S2: qPCR semi-quantitation of monoaminergic transporters, receptors and ADHD associated genes, expressed as min, q1, med, q3, max., in the prefrontal cortex, the parietal cortex, the cerebellum and the striatum of WT, EphA3KI/+ and EphA3KI/KI animals. 5HT1A: serotonin receptor 1A, 5HT1B: serotonin receptor 1B, Adra2A: a-2A adrenergic receptor, Adra2C: a-2C adrenergic receptor, D1: dopaminergic receptor 1, D2: dopaminergic receptor 2, DAT: dopamine transporter, NET: noradrenalin transporter, SNAP25: synaptosomal-associated protein 25.
